# Necrosis of the Upper Jaw from Secondary Syphilis

**Published:** 1867-12

**Authors:** S. D. French


### ARTICLE IV.

### *Necrosis of the Upper Jaw from Secondary Syphilis.*

By S. D. French, D.D.S.

The following case came to me for treatment in the month of February, 1866. The gentleman was a captain in the regular army, and had been stationed at one of the forts in Kansas. He had contracted syphilis while in the army, and had been treated by the surgeon, and cured as he supposed, but after a few months secondary syphilis set in, and was so severe that he was obliged to leave the army and return home. He arrived in October and immediately placed himself under the care of his family physician who treated him till he came to me. Feb. 3d, 1866 he called the first time, and I made a thorough examination of the mouth, and found that the alveolar process around the teeth inclusive of from the first molar on the left around to the second bicuspid on right side (upper maxilla) was necrosed. He informed me that his physician had been attending him since October 25th, and treating him for catarrhal ozena up to the time he called upon me, when, as I said before, I found the bone very much diseased. I made an appointment with him for the next week.

February 9th, patient called according to appointment. I extracted the central, lateral and bicuspid on the right, and the cuspid on the left side, the other teeth having been extracted before. The teeth which I extracted on the right side were very loose, so much so that by pressing the finger against the palatine surface of the teeth you could see up to the apex of the root, the only attachment between the teeth and gums being on the labial surface, and that very slight.

The cuspid on the left side was more firmly attached to the gums. I say gums for the alveolar sockets were so diseased that there was no attachment between them and the teeth. After removing the teeth I commenced the operation of removing the dead bone. I took out some twelve pieces at this sitting, some of them one half an inch square, but the most of them smaller. After I had removed all the dead portions of bone possible at this sitting, I washed the parts with warm water, and then with a weak solution of creosote and iodine equal parts, and gave the patient some wash to use during the day, requesting him to call again at the end of two days. Feb. 12th patient called again. I found that the inflammation had subsided somewhat, but could see that there was considerable of dead bone yet to be removed and with my instruments I commenced operations the second time, to my surprise finding that there was about as much dead bone to be removed as at the former sitting, although the pieces were much smaller. I ordered him to continue the use of the wash. He called again February 14th, when I removed one small piece of dead bone, but aside from the point it occupied, the inflammation had almost entirely disappeared, and the parts had assumed a healthy appearance. I think I never saw such a rapid recovery of diseased parts as in this case. The patient continued the use of the wash for about two weeks longer, at the end of which time I was able to take an impression and make him a substitute for the lost parts, which he has been able to wear without much difficulty. He continued the use of the wash for about six months when the parts were entirely healed. I made a second plate for the same mouth the latter part of June, 1867. I found that new bone had formed, and the mouth was in a healthy condition.*

---

### ARTICLE V.

### *Plan for obtaining a more perfect fit of Vulcanite Plates.*

By W. Leigh Burton, Dentist, Richmond, Va.

(Secretary of the Virginia Dental Society.)

It sometimes happens, after a vulcanite plate has been completed and tried in the mouth, that from some defect

---

*We have received from Dr. French a plaster model of the mouth which shows how successfully he has treated this case. *Editors.*

